# Temporal decomposition of life years lived with disability in India: a growing demographic concern

**DOI:** 10.1186/s12889-019-7057-x

**Published:** 2019-07-19

**Authors:** Kajori Banerjee, Srei Chanda, Laxmi Kant Dwivedi

**Affiliations:** 1 Department of Mathematical Demography and Statistics, International Institute for Population Sciences, Mumbai, India; 2 Department of Population Policies and Programmes, International Institute for Population Sciences, Mumbai, India

## Abstract

**Background:**

To draw optimal benefits of the demographic dividend, healthy life years of the young adults is a growing concern in India. Rising prevalence of chronic non-communicable diseases among the younger population is responsible for increasing the life years lived with disability among them and for affecting their productivity in turn. This study measures the disability burden in various Indian sub-populations and assesses the contribution of disability to the change in person years lived with a disability during 2001–11.

**Methods:**

Data from the Census of India, 2001 and 2011 was used for estimating the age distribution and disability prevalence among males and females. The Sample Registration System was used for age-specific mortality rate to calculate the life table for 15 states in India. Life years Lived with Disability (LLD) were estimated using the Sullivan method. The extension of Arriaga method was used to decompose change in life years lived with disability into Mortality and Disability Effect (ME and DE, respectively). Positive ME explains improvement in life years due to decline in mortality rate and a negative DE explains a decline in disability incidence in 2001–11.

**Results:**

At national level, the disability prevalence has increased from 2001 to 2011. The prevalence of disability and the share of LLD to Life Expectancy (LE) is higher for males. High and medium fertility states scored highest on living with disability to LE ratio and measured DE in the decomposition analysis. At the national level, the DE increased in the age groups of 20–35 years. It was higher among the females. The states that are in the advanced stages of demographic transition show a negative DE.

**Conclusion:**

The study highlights expansion of DE in prime productive years of life, especially among females, in medium and high fertility states. Decline in skilled employment and productivity can be two major economic adversities due to increasing DE in working ages. Disability among young and working age population needs to be prioritised as most of the Indian states stand at crucial stages of demographic transitions.

**Electronic supplementary material:**

The online version of this article (10.1186/s12889-019-7057-x) contains supplementary material, which is available to authorized users.

## Background

The spike in the young and the working age population in India has been a major source of the demographic dividend, which is going to influence the future economic growth of the country. The demographic dividend is defined as the decline in fertility and mortality resulting in bulging of age structure among the younger and working-age population (15–59 years). This age group has immense potential for economic productivity and also reduces the share of dependent in a population [1, 2]. The formative years of a person’s life are associated with wealth building, supporting their families, and securing their future [3]. The health of the working age population is not only a public health concern but is also a major predictor of the demographic dividend being translated into the economic growth of a country. India is expected to reap the demographic dividend through contributions to economic growth by the younger population. States in earlier stages of the demographic transition, like, Bihar, Uttar Pradesh, and Madhya Pradesh have the potential to contribute to more than 30% of the labor supply in India and are projected to be major contributors to the working age population by 2030 [4]. In order to convert the growing working age population into effective labor force, these states need to strengthen their existing health care system and rapidly improve public health outcomes [5, 6].

To maximize the benefits of demographic dividend, healthy life years of the young adults have become a concern. It is of utmost importance to understand the extent of life years lost due to a disability during the economically productive years. India has witnessed a gain of 20 years in life expectancy (LE) at birth since 1970 [7–9], though this number varies across genders, castes, and regions. The share of healthy life years in the LE has been studied previously in reference to the rapid increase in the elderly population [10, 11]. The risk of disability arising from chronic and non-communicable diseases has also been growing among the older population [12–14]. However, in recent times, there has been an expansion of the disease burden due to non-communicable diseases and injuries even in the younger population in India [15, 16]. As much as 60% of all deaths and 23% of premature mortality can be attributed to the rising prevalence of NCDs among the younger population [17]. An empirical study, that measured healthy life expectancy in recent years and made projections for 2020, confirmed that the gain in the healthy life expectancy has been less among the younger population than among the older one [18]. Disability at younger ages can also arise from childhood nutritional deficiencies, developmental disorders, mental retardation, blindness, and other diseases [19, 20]. It leads to a decline in the physical and/or cognitive growth and inhibits participation in the labour force. It is common for the disabled persons to be restricted from participating in active labour due to reduced productivity as a result of their physical, mental limitations or unfavourable environmental factors. In India, the labour participation of the disabled is almost half in comparison to the rest of the working-age population [21, 22] .

Disability prevalence has increased from 2.13 to 2.21% during 2001–11. The share of the disabled population is higher in rural India; however, the rate of increase in the disabled population is higher in the urban areas during the study period (Table 1). The share of the disabled in the population growth of India is heterogeneous. The prevalence of disability has transited more towards the less advanced states like Bihar, Rajasthan, Odisha and Uttar Pradesh [23]. A higher population growth rate in these states has also increased the disability burden [16]. As a result, deprivation has increased, as has exclusion from major economic activities. The absence of proper health care and economic support often debilitates the health conditions of individuals and the development of the household members. Distress financing, resulting from such situations, often pushes households into a vicious cycle of poverty [24]. Evidently, persons with disabilities are marginalized and have a poor standard of living [25]. The extent of marginalization and inclusiveness in the labour market and society have a varying effect across different disabilities [21].

**Table 1:** Prevalence and share of disability by types and sex in India (2001–11)

Panel A: 2001					Panel B: 2011				
		Total	Male	Female			Total	Male	Female
Total	Total	21,906,769	12,605,635	9,301,134	Total	Total	26,814,994	14,988,593	11,826,401
	Share (in total population)	2.13	2.37	1.87		Share (in total population)	2.21	2.40	2.01
Rural	Total	16,388,382	9,410,185	6,978,197	Rural	Total	18,636,358	10,410,559	8,225,799
	Share (in total population)	2.21	2.47	1.93		Share (in total population)	2.24	2.43	2.03
Urban	Total	5,518,387	3,195,450	2,322,937	Urban	Total	8,178,636	4,578,034	3,600,602
	Share (in total population)	1.93	2.12	1.71		Share (in total population)	2.17	2.34	1.98
In seeing	Total	10,634,881	5,732,338	4,902,543	In seeing	Total	5,033,431	2,639,028	2,394,403
	Share (in disabled population)	48.55	45.47	52.71		Share (in disabled population)	18.77	17.61	20.25
In speech	Total	1,640,868	942,095	698,773	In hearing	Total	5,072,914	2,678,584	2,394,330
	Share (in disabled population)	7.49	7.47	7.51		Share (in disabled population)	18.92	17.87	20.25
In hearing	Total	1,261,722	673,797	587,925	In speech	Total	1,998,692	1,122,987	875,705
	Share (in disabled population)	5.76	5.35	6.32		Share (in disabled population)	7.45	7.49	7.40
In movement	Total	6,105,477	3,902,752	2,202,725	In movement	Total	5,436,826	3,370,501	2,066,325
	Share (in disabled population)	27.87	30.96	23.68		Share (in disabled population)	20.28	22.49	17.47
Mental	Total	2,263,821	1,354,653	909,168	Mental Retardation	Total	1,505,964	870,898	635,066
	Share (in disabled population)	10.33	10.75	9.77		Share (in disabled population)	5.62	5.81	5.37
					Mental illness	Total	722,880	415,758	307,122
						Share (in disabled population)	2.70	2.77	2.60
					Others	Total	4,927,589	2,728,125	2,199,464
						Share (in disabled population)	18.38	18.20	18.60
					Multiple disabilities	Total	2,116,698	1,162,712	953,986
						Share (in disabled population)	7.89	7.76	8.07

This study focusses on measuring the impact of disability on the rising LE in India. Disability has not been discussed enough in public health forums. Apart from having socio-economic consequences, disability also influences the extent of healthy life years. The demographic factor plays a pivotal role in determining the health needs of a given population and the priorities to be accorded to a public health issue. Bearing that in mind, this study estimated life years lost due to disability by sex and age. A healthy life is measured through disability-free life expectancies. The present study sheds light on the effect of disability through the demographic lens. The study provides estimates for 15 major states of the country to explain the large regional disparities in India. The decadal decomposition approach assesses the age-wise contribution of disability to the changes in the life years lived with disability for both the sexes. The change in LE was decomposed into mortality and disability effects. The results of the study will provide evidence of different state-specific disability patterns. The estimates can be used as a base to strengthen infrastructure, plan effective resource allocation, design health and financial policies targeted to the disabled population in various states.

## Methods

We used Census of India for the years 2001 and 2011 for the age data of the total population and the disabled population. For constructing the periodic life table, the age-specific mortality rate (ASMR) was taken from the Sample Registration System (SRS) (2001 and 2011). Census is conducted decennially, following an extended de-facto canvasser method. Initially, house listing is done, and then individual enumeration is conducted. Since enumeration of the disabled population can be challenging, measures are taken to understand the signs and symptoms instead of scrutinizing disability simply from the external features.

Measurement of disability in the Census follows the medical model. In 2001, the respondent was directly asked whether he/she had any of the 5 types of disabilities; disability in seeing, in speech, in hearing, in movement, and mental disability. In 2011, the respondent was first asked if he/she was disabled and then asked to classify their disability into 8 categories, viz., in hearing, in seeing, in speech, in movement, mental retardation, mental illness, any others, and multiple disabilities. To address the definitional differences between the two censuses, we considered total disability counts for the two-time points. The details of definitional changes have been mentioned elsewhere [26, 27]. The information on the total population can be obtained from Table C-14 for five-year age group data and from Table C-20 for disability from a socio-cultural section of the Census. The data is given in 5- and 10-year age groups by gender, districts, and states.

SRS is a reliable and valid source of information. It is the main source of continuous information on state-level fertility and mortality, collected through a dual registration system, that is, through a continuous enumeration and through retrospective half-yearly surveys. SRS provides ASMR information for 20 states, of which we used 15 of the bigger states that contribute to almost 90% of the population of India. The Total Fertility Rate (TFR) of 2001 from the “Compendium of India’s Fertility and Mortality Indicators, 1971 – 2013” was used to categorize the states into different levels of fertility. It was used as a proxy for determining the stage of demographic transition at which the states were at the beginning of the study period.

We performed the analysis on 15 states of India in our study. However, the patterns were found to repeat in states with a similar demographic profile. Therefore, we selected 9 states showing unique patterns to analyze the national scenario. The selected states were Uttar Pradesh, Assam, Rajasthan, Punjab, West Bengal, Karnataka, Odisha, Kerala, and Andhra Pradesh. The country-level analysis was done using an aggregate of 15 major states of India. Healthy life expectancy was calculated using Disability-Free Life Expectancy (DFLE) and Life years Lived With Disability (LLD) as given by Sullivan [28]. The Sullivan method is a commonly used life-table based method to estimate health expectancy. It requires fewer data and is easy to comprehend [29]. The Sullivan method gives standard estimates of the true period value of the health expectancy (in this case, disability) when there are regular and smooth transition rates over a long period of time. This fit our data; thus, our estimates derived from this method can be regarded with confidence. The periodic life table was merged with the age-specific disability rates. The disability life expectancy measures – the DFLE and the LLD – were estimated for 2001 and 2011.

The LLD for the two time-points were further decomposed using the extended Arriaga method. The extension of Arriaga method [30] is used to estimate the contribution of mortality and disability to changes in health expectancy in the study decade 2001–2011 [31, 32]. In our study, the Change in Person-Years Lived with Disability (CPYLD) was temporally decomposed into two effects – the Mortality Effect (ME expressed as MOR^i^_x_ in the following expression) and the Disability Effect (DE expressed as DIS^i^_x_ in the following expression) for the age group x to x + i. The decomposition measures the CPYLD due to mortality (ME) and disability (DE) across gender and age groups. A positive ME depicts an increase in the person-years lived if the disability is constant over the study decade. A negative DE depicts a decrease in the proportion of disability in the age group x to (x + i) if person-years lived is kept at a constant.

Mathematically,

$$ \mathrm{CPYLD}=\updelta \left({\pi}_x^i\ast {L}_x^i\right)=\frac{\uppi_{\mathrm{x}\left(\mathrm{t}\right)}^{\mathrm{i}}+{\uppi}_{\mathrm{x}\left(\mathrm{t}+\mathrm{n}\right)}^{\mathrm{i}}}{2}\ast \delta {L}_x^i+\frac{{\mathrm{L}}_{\mathrm{x}\left(\mathrm{t}\right)}^{\mathrm{i}}+{\mathrm{L}}_{\mathrm{x}\left(\mathrm{t}+\mathrm{n}\right)}^{\mathrm{i}}}{2}\ast \delta {\pi}_x^i $$

Where, ${\pi}_x^i$ is the proportion of the disabled in the x to x + i^th^ age group and ${L}_x^i$ is the person life years in the x to x + i ^th^ age group.

$$ \mathrm{iMORx}=\frac{\uppi_{\mathrm{x}\left(\mathrm{t}\right)}^{\mathrm{i}}+{\uppi}_{\mathrm{x}\left(\mathrm{t}+\mathrm{n}\right)}^{\mathrm{i}}}{2}\ast \delta {L}_x^i\kern0.5em \mathrm{iDISx}=\frac{{\mathrm{L}}_{\mathrm{x}\left(\mathrm{t}\right)}^{\mathrm{i}}+{\mathrm{L}}_{\mathrm{x}\left(\mathrm{t}+\mathrm{n}\right)}^{\mathrm{i}}}{2}\ast \delta {\pi}_x^i $$

$$ \updelta \left({\pi}_x^i\ast {L}_x^i\right)={\uppi}_{\mathrm{x}\left(\mathrm{t}+\mathrm{n}\right)}^{\mathrm{i}}\ast {\mathrm{L}}_{\mathrm{x}\left(\mathrm{t}+\mathrm{n}\right)}^{\mathrm{i}}-{\uppi}_{\mathrm{x}\left(\mathrm{t}\right)}^{\mathrm{i}}\ast {\mathrm{L}}_{\mathrm{x}\left(\mathrm{t}\right)}^{\mathrm{i}} $$, where t = 2001 and t + *n* = 2011.

$\delta {L}_x^i$=difference in person years lived in time t and t + n; $\delta {\pi}_x^i$ = difference in the proportion of disability in time t and t + n.

## Results

### Distribution of measures of disability, disability-free life expectancy, and proportion of life expectancy across states

The estimates of LE, DFLE, LLD, and prevalence of disability in 2001 and 2011 are presented in Panel A, B, and C, respectively, in Table 2. At the national level, the LE at birth was found to be 60.04 (2001) and 63.20 (2011) for males; and 61.45 (2001) and 65.72 (2011) for females. The gain in LE at birth was more favorable for females than for males. The DFLE was 58.43 (2001) and 61.49 (2011) for males, while it was 60.10 (2001) and 64.18 (2011) for females. The LLD is measured as the remaining life years lived with disability, turned out to be 1.62 and 1.71 years for males and 1.35 and 1.54 years for females, in 2001 and 2011, respectively. The share of the LLD to the LE was higher for males at the national level. At the national level, the prevalence of disability has increased for both males and females. The rate of increase is higher for females (1.87 to 2.01) than males (2.37 to 2.40).

**Table 2:** Life Expectancies (LE), Disability Free Life Expectancies (DFLE), Life Lived with Disability (LLD), percentage share of DFLE to LE and prevalence of disability across states with different fertility in India over 2001 and 2011

			Panel A: High Fertility States (TFR ≥ 3)											
States	India (3.1)		Uttar Pradesh (4.5)		Bihar (4.4)		Rajasthan (4.0)		Madhya Pradesh (3.9)		Haryana (3.1)		Assam (3.0)	
	2001	2011	2001	2011	2001	2011	2001	2011	2001	2011	2001	2011	2001	2011
Male														
LE	60.04	63.2	58.02	58.75	61.72	64.48	60.19	62.65	56.82	60.47	60.68	63.12	55.70	60.13
DFLE	58.43	61.49	56.48	57.29	59.88	62.66	58.00	60.66	55.05	58.82	58.96	61.42	54.31	58.98
LWD	1.62	1.71	1.54	1.47	1.83	1.82	2.19	1.99	1.77	1.64	1.72	1.70	1.40	1.15
% LWD/LE	2.7	2.71	2.65	2.50	2.97	2.82	3.64	3.18	3.12	2.71	2.83	2.69	2.51	1.91
Prevalence	2.37	2.4	2.37	2.26	2.62	2.47	2.86	2.39	2.62	2.36	2.41	2.34	2.16	1.61
Female														
LE	61.45	65.72	57.31	62.56	61.83	65.17	62.06	65.02	56.68	62.79	62.51	66.94	57.44	62.23
DFLE	60.1	64.18	56.17	61.23	60.49	63.7	60.28	62.89	55.23	61.36	61.06	65.33	56.13	61.03
LWD	1.35	1.54	1.13	1.34	1.34	1.47	1.78	2.13	1.45	1.43	1.45	1.60	1.30	1.20
% LWD/LE	2.2	2.34	1.97	2.14	2.17	2.26	2.87	3.28	2.56	2.28	2.32	2.39	2.26	1.93
Prevalence	1.87	2.01	1.75	1.88	1.90	1.98	2.11	2.17	2.02	1.89	1.85	1.95	1.81	1.46
			Panel B: Medium Fertility States (2.4≤ TFR ≤2.9)											
			Gujarat (2.9)		Orissa (2.6)		Karnataka (2.4)		Punjab (2.4)		Maharashtra (2.4)		West Bengal (2.4)	
			2001	2011	2001	2011	2001	2011	2001	2011	2001	2011	2001	2011
Male														
LE			61.17	63.08	57.21	61.52	60.26	63.12	63.73	65.1	62.22	66.20	62.20	65.64
DFLE			59.53	61.70	55.30	59.33	58.78	61.57	62.38	63.31	60.95	64.13	60.42	63.89
LWD			1.64	1.37	1.91	2.19	1.48	1.55	1.34	1.79	1.27	2.08	1.78	1.74
% LWD/LE			2.68	2.17	3.34	3.56	2.46	2.46	2.10	2.75	2.04	3.14	2.86	2.65
Prevalence			2.29	1.95	3.05	3.18	2.00	2.35	1.95	2.59	1.85	2.91	2.55	2.41
Female														
LE			63.33	66.56	58.87	62.93	64.08	67.56	64.99	69.16	64.10	69.44	64.13	67.56
DFLE			61.94	65.29	57.19	60.92	62.79	66.14	63.91	67.61	63.15	67.70	62.92	66.01
LWD			1.40	1.27	1.68	2.01	1.29	1.42	1.08	1.55	0.95	1.74	1.21	1.55
% LWD/LE			2.21	1.91	2.85	3.19	2.01	2.1	1.66	2.24	1.48	2.51	1.89	2.29
Prevalence			1.81	1.66	2.49	2.74	1.55	1.98	1.51	2.09	1.37	2.35	2.04	2.00
			Panel C: Low Fertility States (TFR< 2.4)											
									Andhra Pradesh (2.3)		Tamil Nadu (2.0)		Kerala (1.8)	
									2001	2011	2001	2011	2001	2011
Male														
LE									59.78	62.66	62.81	65.64	66.47	67.92
DFLE									58.42	60.68	61.16	64.4	64.22	66.16
LWD									1.36	1.98	1.66	1.23	2.25	1.77
% LWD/LE									2.28	3.16	2.64	1.87	3.38	2.61
Prevalence									2.01	2.89	2.52	1.82	2.96	2.46
Female														
LE									63.78	66.60	65.09	68.59	70.76	71.81
DFLE									62.57	64.73	63.19	67.55	68.7	70.17
LWD									1.21	1.87	1.90	1.03	2.06	1.64
% LWD/LE									1.90	2.81	2.92	1.50	2.91	2.28
Prevalence									1.57	2.47	2.74	1.45	2.46	2.11

Note: Total Fertility Rates (TFR) of the states are given in parenthesis beside the state names

The higher fertility states (Table 2, Panel A) showed a higher increase in the population in the age groups 15–59 years and 60 and above years for both the total population and the disabled population in 2001–11 (Additional file 1 and 2). The LE at birth also increased for both the sexes in these states. The highest LE gain was observed in Assam for males (from 55.7 to 60.13) and in Madhya Pradesh for females (56.68 vs. 62.79). The LLD declined for males in all the higher fertility states. By contrast, in the case of females, it increased in most of these states (except Assam and Madhya Pradesh). The highest decline in the LLD was observed in Assam for males (from 1.4 to 1.15). The highest increase in the LLD was observed for females in Rajasthan (from 1.78 to 2.13).

In the medium fertility states (2.4 < TFR < 2.9) (Table 2, Panel B), the LE was found to have increased in all states other than Gujarat. The study observed the highest LE gain in Gujrat for males (from 61.17 to 63.08) and in Maharashtra for females (from 64.1 to 69.44). The LLD increased in most of these states (except for males in West Bengal). The highest increase in the LLD was observed in Maharashtra for both males (from 1.27 to 2.08) and females (from 0.95 to 1.74).

In the low fertility states (TFR < 2.4) (Table 2, Panel C), except Tamil Nadu, the gain in the LE was higher for males than females. The LLD has increased only in Andhra Pradesh. Tamil Nadu and Kerala showed a decline in the LLD for both genders. The share of the population at the working ages was found to be higher for the disabled in most of the states. Additional file 1 and 2 shows that among the working ages, the population increase was higher in the high fertility states than in the medium or the low fertility states. The population growth was higher for the elderly (60 and above age groups) in the low fertility states in 2001–11. The population growth of the disabled was higher in the working ages (15–59 years) in the high and medium fertility states, and in the older ages (60 and above) in the low fertility states in the study duration.

The state-level variations in the increase in the prevalence of disability across gender and those in the share of the LLD to the LE necessitated estimation of the contribution of mortality and disability to the CPYLD in the study decade.

### Decomposition of change in the person-years lived with disability across age and gender during 2001–11

**Fig. 1:**
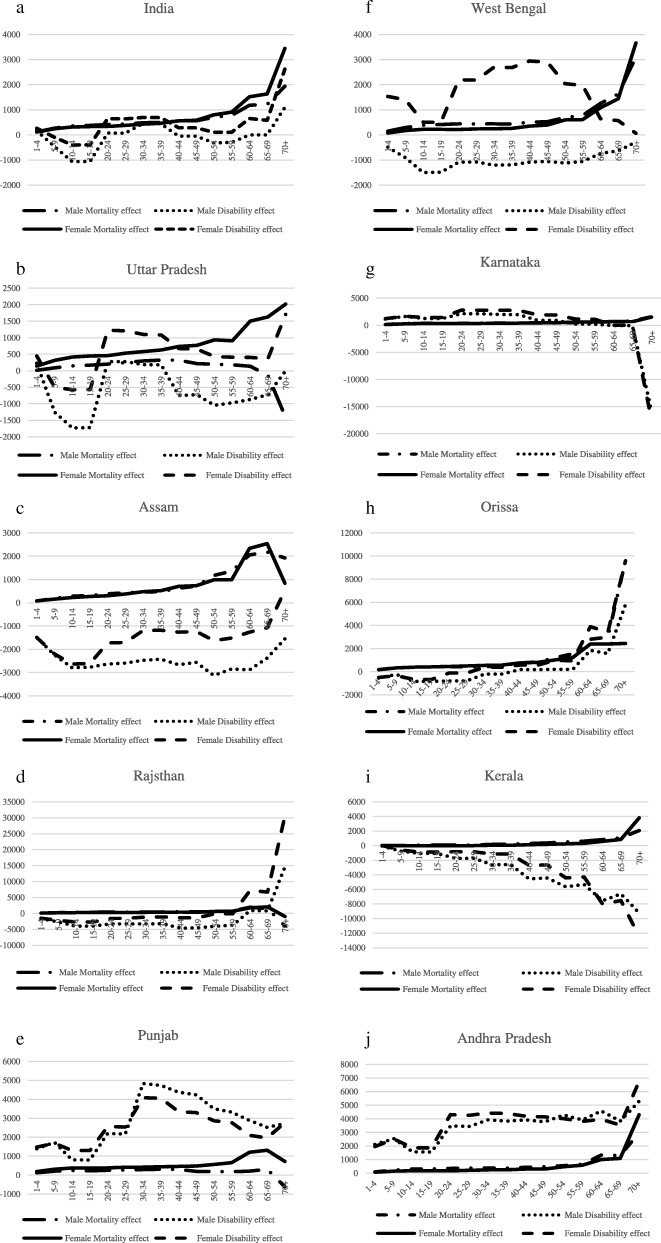
Mortality and Disability Effect (ME and DE) from temporal decomposition of Change in Person Years Lived with Disability (CPYLD) in India and selected states in 2001–2011. Note: x-axis defines age groups y-axis defines mortality and disability effects

Figure 1 (a-j) shows the temporal decomposition of the change in LLD between 2001 and 2011 at the national level and for the selected states. At the national level, the ME was positive for all age groups, implying an increase in the overall LE, with the constraint that the disability had not changed over the study decade. The DE was found to be positive in the working age groups of 20 to 44 years and in the older age groups of 70 and more. This indicates that there was an increase in the LLD due to an increase in disability in these age groups if mortality was held constant over 2001–2011. By contrast, the DE pattern was not homogenous across the states of India.

Figure 1 (b-d) shows the pattern of ME and DE in the higher fertility states. The DE in Uttar Pradesh was positive for females but negative for males in most of the ages. In the working age groups of 20–39 years, the DE was high and positive. In Assam, there were two points of increase in the DE across age groups for both the genders – one in the working age group of 20–24 years and the other in the age group of 60–64 years. The DE for ages below 55 years in Rajasthan was found to lie around the x-axis. It was observed to increase rapidly after reaching the age group of 55–59 years.

Medium fertility states (Fig. 1, e-h) like Punjab and Karnataka showed a positive DE for both genders during 2001–2011. The DE was near the x-axis or was negative for both the genders in Odisha and for males in West Bengal. In Punjab and Karnataka and among the females of West Bengal, the DE was particularly high in the working age groups. This depicts the contribution of disability to the increase in the LLD in these ages.

The low fertility states (Fig. 1, i and j) of Kerala and Andhra Pradesh show a heterogeneous DE pattern. The DE was found to decrease with age for both the genders in Kerala, while the DE was positive for Andhra Pradesh across ages and gender during 2001–11. Andhra Pradesh had two points of increase in DE – one in the age group of 15–19 years, during which it rose and stabilized, and the other in the age group of 65–69 years, from where the DE rose again.

## Discussion

India is projected to have the largest demographic dividend by 2030. To utilize this window of opportunity, evaluation of the healthy life expectancy of the population, with special emphasis on the working age, is required. Our study estimates the changes in the LE, the disability prevalence, and the LLD from 2001 to 2011 in India. To understand the contribution of mortality and disability, we decomposed the CPYLD across age and sex in selected states. The study made two major findings regarding the growth and contribution of disability to LE. We found that disability prevalence increased during 2001–11. The disability and LLD were more prevalent among males than among females. However, females displayed an increase in disability prevalence and the LLD in most of the high and medium fertility states in the study decade. The share of the young and the working age population in the growth of the disabled population was sizeable. Although there was an improvement in the LE, the rise in the occurrence of disability among the working age population increased the LLD. Our second major finding suggests a widespread heterogeneity in the pattern of DE across the selected states in India. In most cases, the DE showed a two-point increase. It first shot up at the younger working ages and, thereafter, stabilized. The second point of the increase was seen in the older ages. The ME is uniformly positive during the study duration due to the rise in the LE.

The key finding of our research highlights a rising burden of disability on the working age groups. In the last few decades, an improvement in child health and mortality in India has directly improved the overall LE and reduced congenital and childhood disability [33, 34]. This suggests that a lesser number of disabled children entered the working age group during the study decade. However, among the working age groups, mainly after 40, there is a spurt in chronic diseases like diabetes, cardiovascular diseases and related disabilities, mental illness and injuries, increasing the burden of diseases [12, 35]. Changes in lifestyle, behavioral changes, rampant urbanization, risky health habits, and substance abuse are the major contributors to the growing burden of diseases [36]. A high incidence of chronic and non-communicable diseases and injuries in younger age groups disfavors LLD and healthy life years too [15, 23, 37]. An early incidence of ill health, along with longer LE, causes prolonged suffering and, in turn, makes the younger people economically underproductive. Unlike among the elderly, disability in the working age groups creates a dual burden in terms of loss of health, which results in high health expenditure, along with a loss of productive employment. Our study finds that increasing DE in the working ages in the major states of India is a cause for concern from the point of view of reaping the demographic dividend in the upcoming decades.

A considerable difference in the LE and the LLD across different states of India is the evidence of differential demographic and epidemiological transitions. In Uttar Pradesh, Bihar, Assam, and other higher fertilty states, the values of DE are relatively elevated for the working age population during 2001-11. This implies that a major proportion of the working age groups bore the disability burden. These laggard states are projected to contribute 50% to the population growth (RGI, 2006) and to make the maximum contribution to the demographic dividend by 2025 [1]. Concerns have arisen as a slower decline in the DE among the working age population is indicative of the chronic nature of diseases in that population. Medium fertility states have already started to realize the dividend with windows of opportunity. Our study shows these states have a positive DE and lower share of the working age population in the total as well as the disabled subgroups. A high and positive value of DE among the working ages in a state like Punjab exemplifies gain in the burden of disability. This is substantiated by a study on age-specific decomposition of LE, whereby the meager change in LE in Punjab was shown to be the result of a growth in mortality among the working ages [34]. Alterations in the dependency ratio due to an unhealthy working age population cause a decline in the income and economic wellbeing. West Bengal has a higher female positive DE, while males show the opposite trend. This trend is supported by the WHO-SAGE (World Health Organization – Study on Global Ageing and Adult Health) survey wave-1 of India, which reported that more females suffer from disability than males in West Bengal [38]. The low fertility states have a higher incidence of disease and disability in the older than working ages. A few low fertility states, such as Kerala and Tamil Nadu, have been successful in limiting the incidence of disability despite the high prevalence of chronic diseases. These states have had a negative DE for both the genders in the last decade. Health outcomes are always associated with good health infrastructure, education, and awareness among the population. Availability of relatively better healthcare services and the prevalence of better health behavior in most of the lower fertility states might be associated with the low LLD, that supports in boosting economic development through active labor participation. [39].

The present study has brought out a gender differential in the disability burden. The DE is found to be higher among the females at the national level and also for some of the selected states. Higher maternal morbidity, nutritional deficiency, neglect, lower decision-making power, and economic dependency could be the reasons for long-term disability among women. A multi-country study claims adult females are more likely than males to report about their poor health and require more care [40]. The regional and gender differentials of disability can be corrected if a comprehensive gender inclusive health care approach is adopted at all levels of health infrastructure.

### Strengths and limitations

The present paper uses data from the Census of India, which collects self-reported and symptom based data on disability in congruence with the medical model of disability. The study design is unable to control the reporting bias that may be present in the data. Though subjectivity of diagnosis of disability remains in doubt, the Census data on disability has several advantages. It is readily available, enumerates the entire population, comparable over time and reliable as it directs trained enumerators to collect data based on detailed definitions considering physical, intellectual or bodily dysfunction. Sample surveys providing data on disability are often incomparable across the surveys, irregular and subjected to sampling errors. The disability indicators were estimated using the total population in 2001 and 2011 to tackle definitional changes. This helped to reduce errors associated with sample surveys. Disability is often associated with stigma, shame, and discrimination. It is common to avoid and marginalize persons with disability in public places [41]. The stigma attached to a disability may lead to under-reporting during enumeration, especially among females. The present study, however, provides an insight into the growing disability burden in India through the demographic lens. The life table assumptions hold valid in the calculation of the LE, the DFLE, and the LLD. Along with the life table assumptions, we also assume that age-specific disability prevalence is constant over time. Under these assumptions, Sullivan’s method is found to provide unbiased and consistent estimates [42]**.** This assumption is not very unrealistic considering that the growth rate of disability in various age-groups is not very drastic yet. The disability proportions were categorized into ten-year age groups. For the purpose of this study, the proportions were equally weighted and divided into five-year age groups. Aggregate disability was taken into account, thereby limiting the study from exploring the burden of different types of disability. The inconsistency of economic, social, and political structures in the different regions of India poses a challenge in the accurate estimation of the LE and the DFLE. The data on disability is limited. The most recent data on disability is found in the Census of India, 2011. However, this data does not specify whether the disability reported is a permanent one or one that can be rectified through medical attention. This study used the Sullivan method to estimate the DFLE, conditional on the assumption that the disabilities reported are permanent in nature. This paper classifies the states on different fertility level as a measure of development. Many states with the same level of fertility have different health infrastructure and parameters. We are limited to include various health indicators like development parameters and infrastructures in this research, however, it can be further extended in future research to measure the association with disability burden.

## Conclusion

This paper documents the rising burden of disability among females and the working age population in India. The states going through the initial stages of the demographic transition bear a higher DE among the working age population. This calls for demand-driven policy and programmatic approaches, depending upon the stage of epidemiological transition of each state. Neglecting health in the early years of life has the potential to degrade DFLE and economic productivity. A reduction in the healthy life years in the young ages can burden the health of the population. This scenario necessitates the healthcare services to be affordable and the labor market to be secure and inclusive.

## Additional file

Additional file 1: Age Distribution for age groups 15-59 and 60+ years for total and disabled population in 2001 and 2011. (DOCX 30 kb)

Additional file 2: Mortality and disability effect (ME and DE) from temporal decomposition of Change in Person Years Lived with Disability (CPYLD) in India and selected states in 2001- 2011. (DOCX 88 kb)

## Data Availability

The study uses data from Census of India and Sample Registration System. This data is available in public domain and can be accessed publicly from http://www.censusindia.gov.in/.

## Abbreviations

ASMR: Age-Specific Mortality Rate
CPYLD: Change in Person-Years Lived with Disability
DE: Disability Effect
DFLE: Disability Free Life Expectancy
LE: Life Expectancy
LLD: Life years Lived with Disability
ME: Mortality Effect
SRS: Sample Registration System
TFR: Total Fertility Rate
