# Supplementary material for: Temporal decomposition of life years lived with disability in India: a growing demographic concern
Source: BMC Public Health. 2019 Jul 19;19:966. doi: 10.1186/s12889-019-7057-x (PMC6642470; doi:10.1186/s12889-019-7057-x)
Supplement: Supplementary file 1 — Age Distribution for age groups 15-59 and 60+ years for total and disabled population in 2001 and 2011. (DOCX 30 kb) [file 12889_2019_7057_MOESM1_ESM.docx]

Additional file 1: Age Distribution for age groups 15-59 and 60+ years for total and disabled population in 2001 and 2011

Table 1.A. Age Distribution for age groups 15-59 and 60+ years for total and disabled population in 2001 and 2011

|  |  |  |  | **High Fertility States (TFR > 3)** | | | | | | | | | | | |
| --- | --- | --- | --- | --- | --- | --- | --- | --- | --- | --- | --- | --- | --- | --- | --- |
|  | **States** | **India (TFR= 3.1)** | | **Uttar Pradesh** | | **Bihar** | | **Rajasthan** | | **Madhya Pradesh** | | **Haryana** | | **Assam** | |
|  |  | **2001** | **2011** | **2001** | **2011** | **2001** | **2011** | **2001** | **2011** | **2001** | **2011** | **2001** | **2011** | **2001** | **2011** |
| **Male** | Age groups |  |  |  |  |  |  |  |  |  |  |  |  |  |  |
| Total | 15-59 | **48.86** | **60.47** | 43.54 | 55.96 | 40.55 | 52.27 | 43.97 | 57.70 | 45.63 | 59.03 | 47.53 | 61.28 | 49.37 | 60.54 |
|  | 60+ | **6.08** | **8.23** | 5.96 | 7.76 | 5.41 | 7.60 | 5.58 | 7.26 | 5.57 | 7.37 | 5.90 | 8.08 | 5.03 | 6.62 |
| Disabled | 15-59 | 58.99 | 61.19 | 54.78 | 59.52 | 54.09 | 56.77 | 57.23 | 53.68 | 57.02 | 60.99 | 57.58 | 61.48 | 58.20 | 59.33 |
|  | 60+ | 15.62 | 18.20 | 13.64 | 15.01 | 12.50 | 14.08 | 17.49 | 28.95 | 17.98 | 18.68 | 18.02 | 19.92 | 15.72 | 20.18 |
| **Female** |  |  |  |  |  |  |  |  |  |  |  |  |  |  |  |
| Total | 15-59 | **48.21** | **60.57** | 43.12 | 56.52 | 41.35 | 52.41 | 43.49 | 57.84 | 44.36 | 58.19 | 46.40 | 61.95 | 47.40 | 60.40 |
|  | 60+ | **6.64** | **9.01** | 5.79 | 7.83 | 5.20 | 7.25 | 6.46 | 8.56 | 6.26 | 8.42 | 6.66 | 9.33 | 4.98 | 6.71 |
| Disabled | 15-59 | 53.72 | 56.13 | 48.39 | 54.71 | 48.64 | 52.34 | 46.27 | 40.82 | 48.99 | 52.48 | 49.04 | 54.71 | 52.70 | 54.99 |
|  | 60+ | 19.53 | 22.63 | 15.58 | 17.39 | 13.25 | 14.19 | 26.22 | 44.02 | 24.79 | 25.37 | 24.95 | 26.67 | 18.25 | 25.10 |
|  |  |  |  | **Medium Fertility States (2.4< TFR <2.9)** | | | | | | | | | | | |
|  |  |  |  | **Gujarat** | | **Odisha** | | **Karnataka** | | **Punjab** | | **Maharashtra** | | **West Bengal** | |
|  |  |  |  | **2001** | **2011** | **2001** | **2011** | **2001** | **2011** | **2001** | **2011** | **2001** | **2011** | **2001** | **2011** |
| **Male** |  |  |  |  |  |  |  |  |  |  |  |  |  |  |  |
| Total | 15-59 |  |  | 50.74 | 63.25 | 51.48 | 61.41 | 52.77 | 64.51 | 52.35 | 63.46 | 52.00 | 63.75 | 53.44 | 64.72 |
|  | 60+ |  |  | 5.19 | 7.16 | 7.10 | 9.43 | 6.22 | 8.88 | 7.61 | 9.88 | 6.78 | 9.05 | 5.96 | 8.24 |
| Disabled | 15-59 |  |  | 62.45 | 64.36 | 57.13 | 56.33 | 60.90 | 64.50 | 59.43 | 64.85 | 60.98 | 64.54 | 62.77 | 64.96 |
|  | 60+ |  |  | 14.75 | 15.01 | 18.92 | 24.64 | 15.17 | 15.31 | 17.12 | 17.48 | 15.89 | 16.15 | 13.31 | 16.63 |
| **Female** |  |  |  |  |  |  |  |  |  |  |  |  |  |  |  |
| Total | 15-59 |  |  | 50.58 | 62.88 | 51.24 | 61.85 | 51.70 | 64.01 | 51.99 | 64.77 | 50.12 | 62.94 | 51.20 | 64.00 |
|  | 60+ |  |  | 6.49 | 8.81 | 7.42 | 9.61 | 7.10 | 10.11 | 8.25 | 10.87 | 8.38 | 10.85 | 6.56 | 8.76 |
| Disabled | 15-59 |  |  | 54.66 | 59.02 | 53.51 | 52.91 | 54.43 | 61.36 | 54.03 | 61.74 | 53.94 | 60.26 | 56.99 | 60.70 |
|  | 60+ |  |  | 21.95 | 20.92 | 21.56 | 28.67 | 19.92 | 18.19 | 21.13 | 20.20 | 20.48 | 19.19 | 16.21 | 20.11 |
|  |  |  |  | **Low Fertility States (TFR< 2.4)** | | | | | | | | | | | |
|  |  |  |  |  |  |  |  |  |  | **Andhra Pradesh** | | **Tamil Nadu** | | **Kerala** | |
|  |  |  |  |  |  |  |  |  |  | **2001** | **2011** | **2001** | **2011** | **2001** | **2011** |
| **Male** |  |  |  |  |  |  |  |  |  |  |  |  |  |  |  |
| Total | 15-59 |  |  |  |  |  |  |  |  | 55.20 | 64.07 | 55.01 | 65.61 | 60.84 | 63.32 |
|  | 60+ |  |  |  |  |  |  |  |  | 6.56 | 9.29 | 7.58 | 10.14 | 9.27 | 11.77 |
| Disabled | 15-59 |  |  |  |  |  |  |  |  | 60.62 | 62.62 | 66.85 | 68.00 | 63.24 | 62.47 |
|  | 60+ |  |  |  |  |  |  |  |  | 17.10 | 20.40 | 13.90 | 15.81 | 25.06 | 25.69 |
| **Female** |  |  |  |  |  |  |  |  |  |  |  |  |  |  |  |
| Total | 15-59 |  |  |  |  |  |  |  |  | 54.31 | 64.18 | 55.31 | 66.36 | 60.14 | 64.57 |
|  | 60+ |  |  |  |  |  |  |  |  | 7.26 | 10.47 | 7.70 | 10.70 | 10.66 | 13.30 |
| Disabled | 15-59 |  |  |  |  |  |  |  |  | 54.04 | 59.51 | 71.85 | 67.42 | 55.15 | 56.42 |
|  | 60+ |  |  |  |  |  |  |  |  | 22.53 | 23.77 | 13.07 | 16.56 | 34.52 | 33.77 |
