# Supplementary material for: Temporal decomposition of life years lived with disability in India: a growing demographic concern
Source: BMC Public Health. 2019 Jul 19;19:966. doi: 10.1186/s12889-019-7057-x (PMC6642470; doi:10.1186/s12889-019-7057-x)
Supplement: Supplementary file 2 — Mortality and disability effect (ME and DE) from temporal decomposition of Change in Person Years Lived with Disability (CPYLD) in India and selected states in 2001- 2011. (DOCX 88 kb) [file 12889_2019_7057_MOESM2_ESM.docx]

Additional file 2: Mortality and disability effect (ME and DE) from temporal decomposition of Change in Person Years Lived with Disability (CPYLD) in India and selected states in 2001- 2011

Figure 1.A. Mortality and disability effect (ME and DE) from temporal decomposition of Change in Person Years Lived with Disability (CPYLD) in India and selected states in 2001- 2011
